# Development and validation of a simple-to-use nomogram to predict the deterioration and survival of patients with COVID-19

**DOI:** 10.1186/s12879-021-06065-z

**Published:** 2021-04-16

**Authors:** Zhiyong Zeng, Chaohui Wu, Zhenlv Lin, Yong Ye, Shaodan Feng, Yingying Fang, Yanmei Huang, Minhua Li, Debing Du, Gongping Chen, Dezhi Kang

**Affiliations:** 1 Department of Hematology, The First Affiliated Hospital of Fujian Medical University, Fuzhou, 350005 China; 2 Department of Pharmacy, The First Affiliated Hospital of Fujian Medical University, Fuzhou, China; 3 Department of Emergency, The First Affiliated Hospital of Fujian Medical University, Fuzhou, China; 4 Department of Intensive Care Unit, Fujian Provincial Cancer Hospital, Fuzhou, China; 5 Department of Tuberculosis, the Third People’s Hospital of Yichang, Yichang, China; 6 Department of Respiratory and Critical Care Medicine, The First Affiliated Hospital of Fujian Medical University, Fuzhou, 350005 China; 7 Department of Neurosurgery, The First Affiliated Hospital of Fujian Medical University, Fuzhou, 350005 China

**Keywords:** Nomogram, COVID-19, Prediction, Deterioration, Survival

## Abstract

**Background:**

COVID-19 pandemic has forced physicians to quickly determine the patient’s condition and choose treatment strategies. This study aimed to build and validate a simple tool that can quickly predict the deterioration and survival of COVID-19 patients.

**Methods:**

A total of 351 COVID-19 patients admitted to the Third People’s Hospital of Yichang between 9 January to 25 March 2020 were retrospectively analyzed. Patients were randomly grouped into training (*n* = 246) or a validation (*n* = 105) dataset. Risk factors associated with deterioration were identified using univariate logistic regression and least absolute shrinkage and selection operator (LASSO) regression. The factors were then incorporated into the nomogram. Kaplan-Meier analysis was used to compare the survival of patients between the low- and high-risk groups divided by the cut-off point.

**Results:**

The least absolute shrinkage and selection operator (LASSO) regression was used to construct the nomogram via four parameters (white blood cells, C-reactive protein, lymphocyte≥0.8 × 10^9^/L, and lactate dehydrogenase ≥400 U/L). The nomogram showed good discriminative performance with the area under the receiver operating characteristic (AUROC) of 0.945 (95% confidence interval: 0.91–0.98), and good calibration (*P* = 0.539). Besides, the nomogram showed good discrimination performance and good calibration in the validation and total cohorts (AUROC = 0.979 and AUROC = 0.954, respectively). Decision curve analysis demonstrated that the model had clinical application value. Kaplan-Meier analysis illustrated that low-risk patients had a significantly higher 8-week survival rate than those in the high-risk group (100% vs 71.41% and *P* < 0.0001).

**Conclusion:**

A simple-to-use nomogram with excellent performance in predicting deterioration risk and survival of COVID-19 patients was developed and validated. However, it is necessary to verify this nomogram using a large-scale multicenter study.

**Supplementary Information:**

The online version contains supplementary material available at 10.1186/s12879-021-06065-z.

## Introduction

The respiratory disease coronavirus disease 2019 (COVID-19) caused by severe acute respiratory coronavirus type 2 (SARS-CoV-2) has been spreading globally since December 2019 [1, 2]. COVID-19 information is becoming more detailed as clinical cases increase. Most patients can be cured clinically. However, some COVID-19 patients get worse due to progressive pneumonia, severe dyspnea, gastrointestinal bleeding, or multiple organ failure, and even die [3–5]. Critically ill patients are at a higher risk of death, with a mortality rate of up to 49% [3]. Currently, there are no drugs for the COVID-19 treatment [6–9]. Therefore, it is urgent to determine factors to quickly predict the deterioration of COVID-19 patients.

Cheng et al. [10] showed that the MuLBSTA score (the multilobular infiltration, hypolymphocytosis, bacterial coinfection, smoking history, hypertension, and age) can be used to predict COVID-19 pneumonia mortality. However, in this study, only two of the 11 deaths were consistent with the MuLBSTA score. Furthermore, there is a discrepancy between the disease severity and the MuLBSTA score in clinical trials. The disease can rapidly spread in some patients with a low MuLBSTA score. Ji et al. [11] showed that CALL Score (comorbidity, age, lymphocyte, and lactate dehydrogenase) can be used to predict progression risk in COVID-19 patients. Liang et al. [12] reported that clinical risk score (chest radiographic abnormality, age, hemoptysis, dyspnea, unconsciousness, comorbidities, cancer history, neutrophil-to-lymphocyte ratio, lactate dehydrogenase, and direct bilirubin) is associated with critical illness. However, these methods are complex and require professional radiologists and respiratory doctors to assess the infiltration of multiple lung lobes, especially during this time of the severe epidemic.

Therefore, this study aimed to construct a simple nomogram using common clinical features for the early identification of COVID-19 patients with rapid deterioration and help clinicians to quickly choose better treatment strategies.

## Methods

### Study participants

The consecutive COVID-19 patients were those admitted to Third People’s Hospital of Yichang between 9 January to 25 March 2020. This study included only patients with positive fluorescence reverse transcription-polymerase chain reaction (RT-PCR) assay results of nasal and pharyngeal swab specimens or specific IgM or IgG antibodies in serum. The disease condition at hospital admission was evaluated and classified into four groups (Mild, Moderate, Severe, and Critical) according to the “COVID-19 diagnosis and treatment program” (7th version) issued by the National Health Commission of China [13]. Patients were reclassified if their condition deteriorated and reached a higher classification criterion.

Disease deterioration was the primary outcome, defined as a change in disease severity from Mild to Moderate/Severe/Critical, Moderate to Severe/Critical, or Severe to Critical during hospitalization [13]. Mortality was the secondary outcome during the treatment.

### Candidate predictors

Candidate variables such as demographics (age, gender, and smoking history), comorbid conditions (diabetes mellitus (DM), hypertension, coronary heart disease (CHD), cardiovascular disease (CVD), chronic obstructive pulmonary disease (COPD), cancer, immunodeficiency) were obtained from electronic medical records. Besides laboratory variables were obtained from blood. Whole blood count, coagulation function, routine biochemistry, C-reactive protein (CRP), and procalcitonin (PCT) were assessed in the lab.

### Statistical analysis

Continuous normal distribution was expressed as mean ± standard deviation (SD). However, non-continuous normal distribution was expressed as median and interquartile (IQR). Categorical variables were expressed using numbers and proportion (%). The total cohort was randomly divided into training and validation cohorts (7:3). A total of 246 (70.0%) patients were in the training dataset and 105 (30.0%) in the validation dataset. The risk model was established using the following three steps to choose the best predictors of disease deterioration. First, candidate predictors with significant *P* values were selected using the univariate logistic regression analysis. The optimum subset of predictors was then screened using the least absolute shrinkage and selection operator (LASSO) regression analysis [14]. The lambda parameter that minimized expected model deviance was selected. Finally, the nomogram was constructed using the coefficients for each predictor provided via LASSO regression.

The area under the curve (AUC) of receiver operating characteristic (ROC) analysis was used to evaluate the discriminative performance of the nomogram. A calibration curve was used to measure the nomogram calibration. The Hosmer-Lemeshow test was used to examine the goodness-of-fit [15]. Moreover, the clinical usefulness of the nomogram was assessed using decision curve analysis (DCA), which quantified net benefits at various threshold probabilities [16, 17]. The net benefit was determined by subtracting the false-positive patients from true-positive patients, weighting by the relative harm of no treatment against the negative effects of unnecessary treatment. True positives were the unit of net benefit. For instance, a net benefit of 0.07, means “7 true positives for every 100 patients in the target population.” The decision curves showed that the nomogram is more beneficial than treating either all or no patients, indicating that the nomogram is clinically useful. The performance of the nomogram was further validated in the validation and total cohorts using the earlier described method. Statistics software SPSS 20.0 (IBM, Chicago, IL)) and R software version 3.6.0 were used for all data analysis. A two-sided *P* < 0.05 indicated a significant difference.

## Results

### Patient characteristics

A total of 360 consecutive records were included. Nine records were excluded, of which six were duplicate records and three did not have laboratory data. Finally, 351 COVID-19 patients met the study requirements. The random number table was used to randomly divide the total cohort into training cohort (*n* = 246, 70%) and validation cohort (*n* = 105, 30%). The clinical characteristics of the training and validation cohorts are shown in Table 1. Despite continuous renal replacement therapy (CRRT), baseline characteristics of both cohorts were comparable, indicating that they could be used as training and validation cohorts.

**Table 1:** Clinical characteristics of patients infected with COVID-19

Characteristics	Total(***n*** = 351)	Training dataset (***n*** = 246)	Validation dataset (***n*** = 105)	***P*** value^**a**^
**Demographics**				
Age (years), median (IQR)	54(38–66)	54(38–66.25)	54(37–65.5)	0.581
Female sex, no. (%)	162(46.2)	117(47.6)	45(42.9)	0.418
Smoking history, *n* (%)	57(16.2)	41(16.7)	16(15.2)	0.74
**Comorbid conditions,** ***n*** **(%)**				
Hypertension	80(22.8)	53(21.5)	27(25.7)	0.407
Diabetes mellitus	41(11.7)	27(11.0)	14(13.3)	0.529
Coronary heart disease	20(5.7)	13(5.3)	7(6.7)	0.609
Cerebrovascular diseases	13(3.7)	9(3.7)	4(3.8)	0.922
COPD	9(2.6)	7(2.8)	2(1.9)	0.73
Cancer	9(2.6)	9(3.7)	0(0)	0.062
Immunodeficiency	1(0.3)	1(0.4)	0(0)	1
**Laboratory data**				
White blood cells (×10^9^/L), median (IQR)	6.3(5.2–8.4)	6.35(5.2–8.5)	6.2(5.25–8.05)	0.725
Neutrophil (×10^9^/L), median (IQR)	4.21(3.22–6.34)	4.215(3.2325–6.6425)	4.17(3.145–6.135)	0.696
Monocyte (×10^9^/L), median (IQR)	0.21(0.15–0.28)	0.21(0.14–0.28)	0.21(0.15–0.27)	0.953
Lymphocyte (×10^9^/L), median (IQR)	0.91(0.62–1.33)	0.91(0.5975–1.3325)	0.9(0.675–1.345)	0.412
Distribution, no. (%)				0.85
< 0.8 (×10^9^/L)	133(37.9)	94(38.2)	39(37.1)	
≥ 0.8 (×10^9^/L)	218(62.1)	152(61.8)	66(62.9)	
Hemoglobin (g/L), median (IQR)	108(98–121)	107(97.75–120)	110(100–122)	0.365
Platelet (×10^9^/L), median (IQR)	130(98–170)	130(98–169.25)	130.5(96.25–171.5)	0.734
PT (s), median (IQR)	10.9(10.5–11.3)	10.9(10.5–11.3)	10.8(10.4–11.2)	0.157
APTT (s), median (IQR)	29.7(26.3–33.6)	29.85(26.8–33.575)	29.25(25.425–33.8)	0.284
Fibrinogen (g/L)	2.664(2.026–3.645)	2.664(2.026–3.542)	2.975(2.081–3.959)	0.192
D-Dimer (mg/L), median (IQR)	0.6(0.51–1.365)	0.62(0.52–1.518)	0.57(0.51–1.048)	0.052
TBIL (μmol/L), median (IQR)	9.19(6.63–13.77)	9.205(6.635–14.015)	8.99(6.745–13.385)	0.869
DBIL (μmol/L), median (IQR)	2.39(1.585–3.57)	2.41(1.6225–3.5375)	2.35(1.56–3.845)	0.866
Albumin (g/L), median (IQR)	37.4(34–40.9)	37.1(34–40.6)	38.1(34–41.3)	0.223
Globulin (g/L), median (IQR)	26.4(23.825–28.8)	26.4(23.725–28.875)	26.25(23.925–28.75)	0.907
ALT (U/L), median (IQR)	21(13.75–34)	20(14–34.5)	21(13–33)	0.69
AST (U/L), median (IQR)	21(16–28)	21(16–29)	22(17–28)	0.786
ALT peak (U/L), median (IQR)	35(24–61)	35(24–61)	34(22–61.5)	0.984
AST peak (U/L), median (IQR)	26(20–39)	26(20–42)	25(20–38)	0.432
Creatinine (μmol/L), median (IQR)	67.35(54.1–79.825)	67.25(54.65–80.35)	68.2(53.65–79.5)	0.57
Creatinine peak (μmol/L), median (IQR)	73.4(58.25–87.925)	73.1(58.2–87.75)	74(58.25–88.65)	0.993
LDH (U/L), median (IQR)	207(164.75–263)	203(164.25–269.5)	211.5(166–260.75)	0.882
LDH peak (U/L), median (IQR)	224.5(175–305.25)	225(175–311.5)	220(177.5–292)	0.762
Distribution, no. (%)				0.92
< 400 (U/L)	286(81.5)	200(81.3)	86(81.9)	
≥ 400 (U/L)	40(11.4)	29(11.8)	11(10.5)	
NA	25(7.1)	17(6.9)	8(7.6)	
CK (U/L), median (IQR)	63(41–111.5)	58.5(40–108.5)	69.5(43–126.75)	0.126
CK-MB (U/L), median (IQR)	12.1(9.4–17.7)	12.1(9.5–17.5)	12.2(9.33–18.48)	0.777
CRP (mg/L), median (IQR)	21.2(4.65–51.625)	20.7(4.35–52.475)	21.55(6.3–48.475)	0.764
PCT (ng/L), median (IQR)	0.08(0.05–0.135)	0.08(0.05–0.1375)	0.08(0.06–0.135)	0.665
**Clinical classification, no. (%)**				0.162
Mild	6(1.7)	2(0.8)	4(3.8)	
Moderate	279(79.5)	195(79.3)	84(80)	
Severe	33(9.4)	26(10.6)	7(6.7)	
Critical	33(9.4)	23(9.3)	10(9.5)	
**Treatment, no. (%)**				
Antiviral treatment	349(99.4)	244(99.2)	105(100)	1
Antibacterial treatment	328(93.4)	229(93.1)	99(94.3)	0.678
Antifungal treatment	20(5.7)	13(5.3)	7(6.7)	0.33
Glucocorticoids	119(33.9)	85(34.6)	34(32.4)	0.365
Intravenous immunoglobulin therapy	66(18.8)	46(18.7)	20(19.0)	0.39
CRRT	5(1.4)	3(1.2)	2(1.9)	0.024^b^
NIVV or high-flow nasal cannula	37(10.5)	28(11.4)	9(8.6)	0.432
Invasive mechanical ventilation	12(3.4)	7(2.8)	5(4.8)	0.354
Aggravation, no. (%)				0.905
Mild to Moderate/Severe/Critical	0(0)	0(0)	0(0)	
Moderate to Severe/Critical	19(5.4)	14(5.7)	5(4.8)	
Severe to Critical	31(8.8)	21(8.5)	10(9.5)	
No	301(85.8)	211(85.8)	90(85.7)	
Death, no. (%)	14(4.0)	9(3.7)	5(4.8)	0.766

Notes: *IQR* interquartile, *COPD* chronic obstructive pulmonary disease, *PT* prothrombin time, *APTT* activated partial thromboplastin time, *TBIL* total bilirubin, *DBIL* direct bilirubin, *ALT* alanine aminotransferase, *AST* aspartate Aminotransferase, *LDH* lactate dehydrogenase, *CK* creatine kinase, *CKMB* Creatine kinase-MB, *CRP* C-reactive protein, *PCT* procalcitonin, *CRRT* continuous renal replacement therapy, *NIVV* non-invasive ventilation, *NA* not available; ^a^For comparison between training dataset and validation dataset; ^b^*P* < 0.05

### Selection of predicting factors associated with deterioration risk

**Fig. 1:**
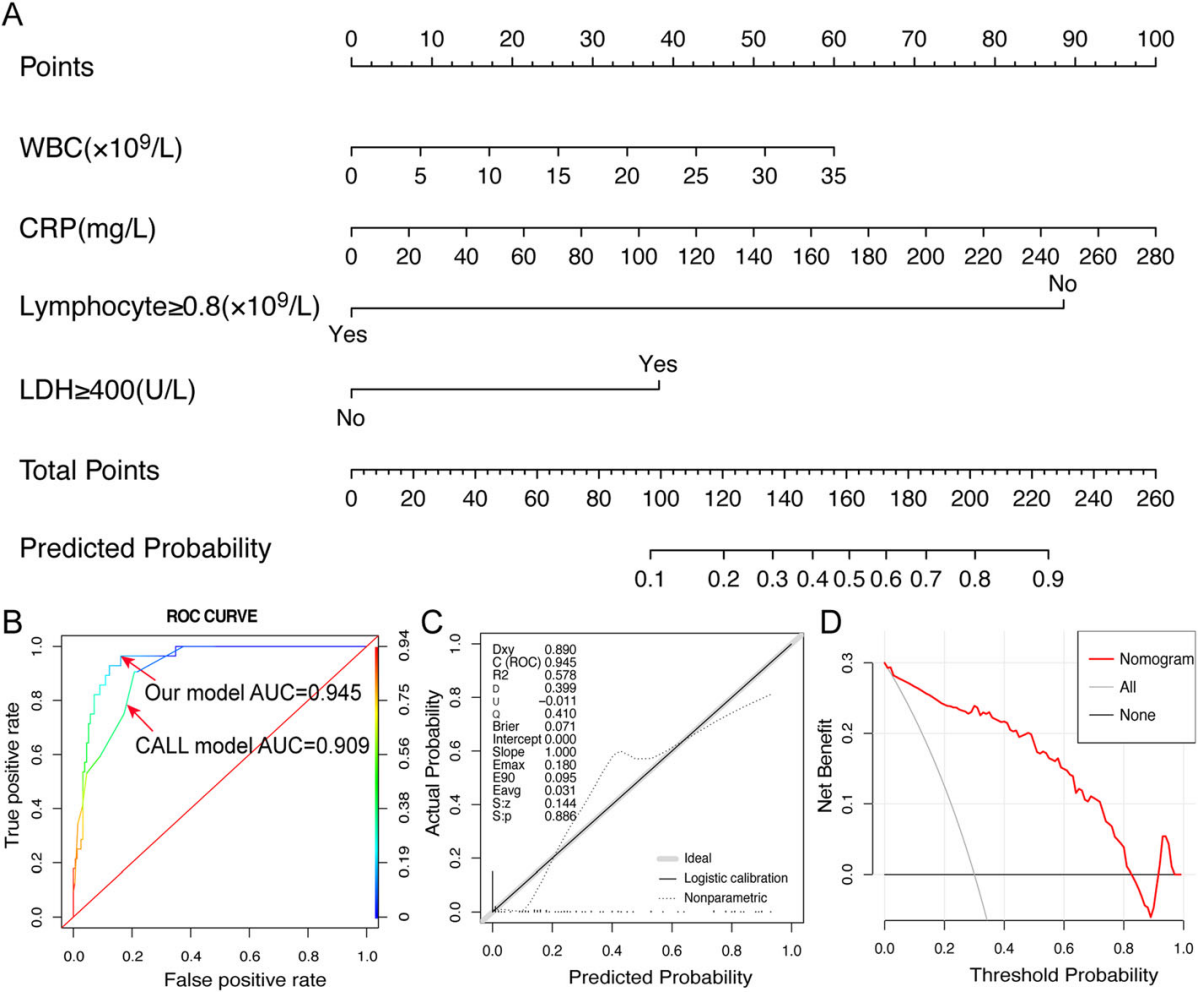
Nomogram predicting the deterioration risk of COVID-19 patients in the training dataset. **a** Nomogram; **b** ROC curve; AUC of 0.945; **c** Calibration plot; **d** DCA. The y-axis represents the net benefit, the x-axis represents the threshold probability. The red line represents the nomogram, and gray and black lines represent the deteriorated patients and patients without deterioration, respectively. Abbreviations: COVID-19, coronavirus disease 2019; ROC, receiver operating characteristic; AUC, area under the curve; DCA, decision curve analysis

A total of 35 potential predicting factors were used in the development stage of the model. Univariate logistic analysis of the training cohort showed that 24 factors, including age, hypertension, DM, hypertension, CHD, CVD, cancer, White blood cells (WBC), Neutrophil (N), Hemoglobin (HB), Monocyte (Mono), whether lymphocyte (Lym) ≥ 0.8 × 10^9^/L, Platelet (PLT), prothrombin time (PT), Fibrinogen (Fib), D-Dimer, lactate dehydrogenase (LDH) ≥ 400 U/L, aspartate Aminotransferase (AST), alanine aminotransferase (ALT), ASTpeak, ALTpeak, Albumin (ALB), Creatinine, Creatinine peak, CRP, and PCT were significantly associated with deterioration risk (Table 2). LASSO regression was used to build the model since the sample size in this study was inadequate to satisfy the recommended guide of events per variable [18]. The optimal tuning parameter (λ) value of 0.07 with log(λ) = − 2.659 was selected (the minimum criteria). In the training cohort, 24 relevant variables were reduced to four potential predictors (Fig. 1S). The four variables (WBC, CRP, Lym ≥ 0.8 × 10^9^/L, LDH ≥ 400 U/L) with non-zero coefficients were presented in the final model (Table 3).

**Table 2:** Univariate logistic regression of progression factors in patients with COVID-19

Variables	OR	95CI%	Estimate	S. E	z value	***P*** value
Age (years)	1.339	1.191–1.498	0.041	0.012	3.399	0.001^b^
Sex						
Male	1	–	–	–	–	–
Female	0.699	0.337–1.448	−0.358	0.372	−0.964	0.335
Smoking	1.598	0.668–3.823	0.469	0.445	1.054	0.292
Hypertension	3.96	1.864–8.415	1.376	0.385	3.579	3.452E-04^b^
Diabetes mellitus	5.586	2.324–13.426	1.720	0.447	3.845	1.207E-04^b^
Coronary heart disease	8.542	2.678–27.24	2.145	0.592	3.625	2.889E-04^b^
Cerebrovascular diseases	5.29	1.347–20.777	1.646	0.698	2.358	0.018^b^
COPD	2.497	0.465–13.404	0.915	0.857	1.067	0.286
Cancer	8.625	2.193–33.922	2.155	0.699	3.084	0.002^b^
Immunodeficiency	1.00E+ 10	0	16.392	882.743	0.019	0.985
White blood cells	1.339	1.197–1.498	0.292	0.057	5.116	3.120E-07^b^
Neutrophil	1.225	1.12–1.34	0.203	0.046	4.423	9.740E-06^b^
Monocyte	0	0–0.027	−7.771	2.122	−3.662	2.507E-04^b^
Lymphocyte						
< 0.8(×10^9^/L)	1	–	–	–	–	
≥ 0.8(×10^9^/L)	0.012	0.002–0.087	−4.449	1.026	−4.337	1.440E-05^b^
Hemoglobin (g/L)	0.942	0.92–0.965	−0.060	0.012	−4.969	6.740E-07^b^
Platelet (×10^9^/L)	0.984	0.976–0.993	−0.016	0.004	−3.627	2.868E-04^b^
PT (s)	1.785	1.335–2.387	0.580	0.148	3.912	9.140E-05^b^
APTT (s)	1.05	0.994–1.108	0.049	0.028	1.747	0.081
Fibrinogen (g/L)	0.542	0.363–0.81	−0.612	0.205	−2.990	0.003^b^
D-Dimer (mg/L)	1.114	1.059–1.171	0.108	0.026	4.201	2.650E-05^b^
TBIL (μmol/L)	1.018	0.963–1.075	0.017	0.028	0.624	0.533
DBIL (μmol/L)	1.023	0.983–1.065	0.023	0.021	1.129	0.259
Albumin (g/L)	0.876	0.819–0.938	−0.132	0.035	−3.814	1.369E-04^b^
Globulin (g/L)	1.016	0.932–1.108	0.016	0.044	0.362	0.717
ALT (U/L)	1.011	0.997–1.025	0.011	0.007	1.553	0.120^b^
AST (U/L)	1.032	1.011–1.054	0.032	0.011	2.954	0.003^b^
ALT peak (U/L)	1.005	1.001–1.009	0.005	0.002	2.400	0.016^b^
AST peak (U/L)	1.007	1–1.013	0.007	0.003	1.968	0.049^b^
Creatinine (μmol/L)	1.007	1–1.013	0.007	0.003	2.057	0.040^b^
Creatinine peak (μmol/L)	1.01	1.003–1.016	0.010	0.003	2.953	0.003^b^
CK (U/L)	1.001	1–1.003	0.001	0.001	1.445	0.148
CKMB (U/L)	1.005	0.999–1.012	0.005	0.003	1.621	0.105
LDH (U/L)						
< 400 U/L	1	–	–	–	–	–
≥ 400 U/L	17.472	7.053–43.284	2.861	0.463	6.180	6.390E-10^b^
CRP (mg/L)	1.029	1.02–1.039	0.029	0.004	6.468	9.960E-11^b^
PCT (ng/L)	1.107	1.031–1.189	0.102	0.036	2.793	0.005^b^

Notes: *OR* odds ratio, *CI* confidence interval, *S. E* standard error, *COPD* chronic obstructive pulmonary disease, *PT* prothrombin time, *APTT* activated partial thromboplastin time, *TBIL* total bilirubin, *DBIL* direct bilirubin, *ALT* alanine aminotransferase, *AST* aspartate Aminotransferase, *LDH* lactate dehydrogenase, *CK* creatine kinase, *CKMB* Creatine kinase-MB, *CRP* C-reactive protein, *PCT* procalcitonin, *NA* not available; ^b^*P* < 0.05

**Table 3:** Risk factors for disease deterioration of patients with COVID-19 identified by LASSO regression

Intercept and variable	LASSO coefficient	Regression coefficient (β)
Intercept	−2.298	−4.262
WBC	0.025	0.059
CRP	0.008	0.012
Lym ≥ 0.8 × 10^9^/L	0.977	3.056
LDH ≥ 400 U/L	−0.759	−1.3

Notes: *LASSO* least absolute shrinkage and selection operator, *WBC* white blood cells, *CRP* C-reactive protein, *Lym* lymphocyte, *LDH* lactate dehydrogenase

### Prediction nomogram development in the training cohort

A nomogram incorporating the above four independent predictive factors was built (Fig. 1a). AUC of the ROC curve was calculated to assess the performance comparison between this nomogram and the published CALL model [11] (comorbidity, age, lymphocyte, and LDH). The AUC of this nomogram in the training cohort was 0.945, which was higher than the AUC of the CALL model (AUC = 0.909) (Fig. 1b). The cut-off value for risk probability in this model was 0.188, with a sensitivity and specificity of 87.7 and 92.9%, respectively. The calibration curve of this nomogram for the deterioration risk in the training cohort between the observed and predicted risks was consistent (Fig. 1c). The non-significant Hosmer-Lemeshow test (Chi-square = 7.951, *P*-value = 0.539) indicated a good fit to the model. The clinical value of the nomogram was evaluated using the DCA analysis (Fig. 1d). The DCA curve indicated that if the threshold probability of a patient was between 30 to 80%, using this nomogram to predict patients who could deteriorate is more beneficial than using the “treat-all” or the “treat-no” schemes.

### Validation of nomogram performance in the validation and total cohorts

**Fig. 2:**
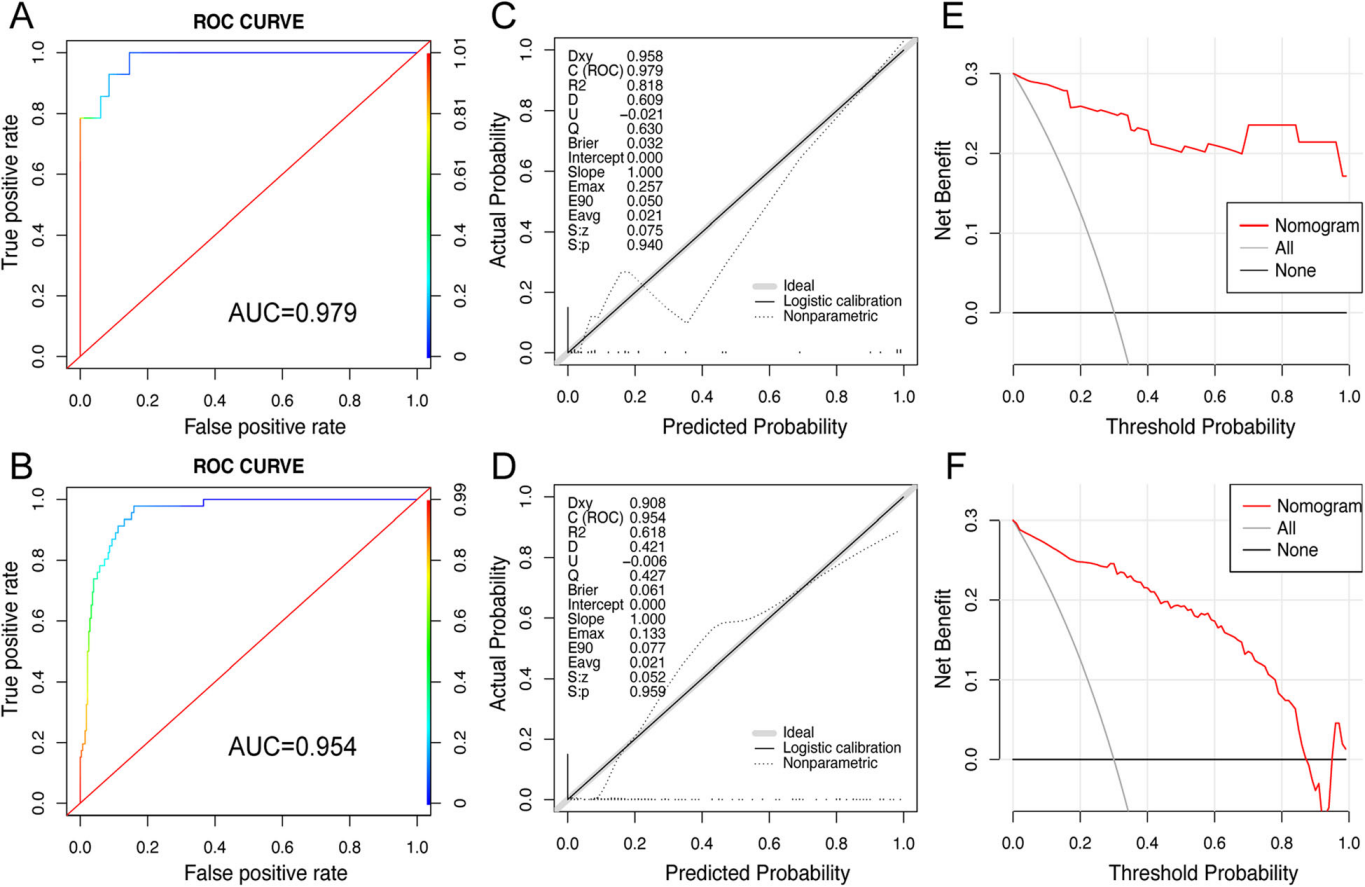
Validation of the discrimination power of the nomogram in the validation and total cohorts. **a-b** ROC curve analysis of the nomogram in the validation and the total cohorts (AUC, 0.979 and 0.954, respectively); **c-d** Calibration plot of the nomogram in the validation and the total cohorts; **e-f** DCA analysis of the nomogram in the validation and the total cohorts. The y-axis represents the net benefit, the x-axis represents the threshold probability. The red line represents the nomogram, and gray and black lines represent the deteriorated patients and patients without deterioration, respectively. Abbreviations: ROC, receiver operating characteristic; AUC, area under the curve; DCA, decision curve analysis

The accuracy of the nomogram in predicting deterioration of COVID-19 patients was high in the validation and total cohorts (AUC = 0.979 and AUC = 0.954, respectively; Fig. 2a-b). Furthermore, calibration plots suggested reasonably good calibration in the validation and total cohorts. The internal calibration plots indicated substantial consistency between the risk predicted by the nomogram and the observed deterioration (Fig. 2c-d). The Hosmer–Lemeshow tests exhibited no statistical significance (Chi-square = 2.172, *P*-value = 0.988; and Chi-square = 6.577, *P*-value = 0.681; respectively), suggesting good fitting of the nomogram. Besides, DCA analysis demonstrated significant positive net benefits in the predictive nomogram, exhibiting the favorable potential clinical effect of the nomogram (Fig. 2e-f).

### Nomogram for predicting the disease severity and the survival of COVID-19 patients

**Fig. 3:**
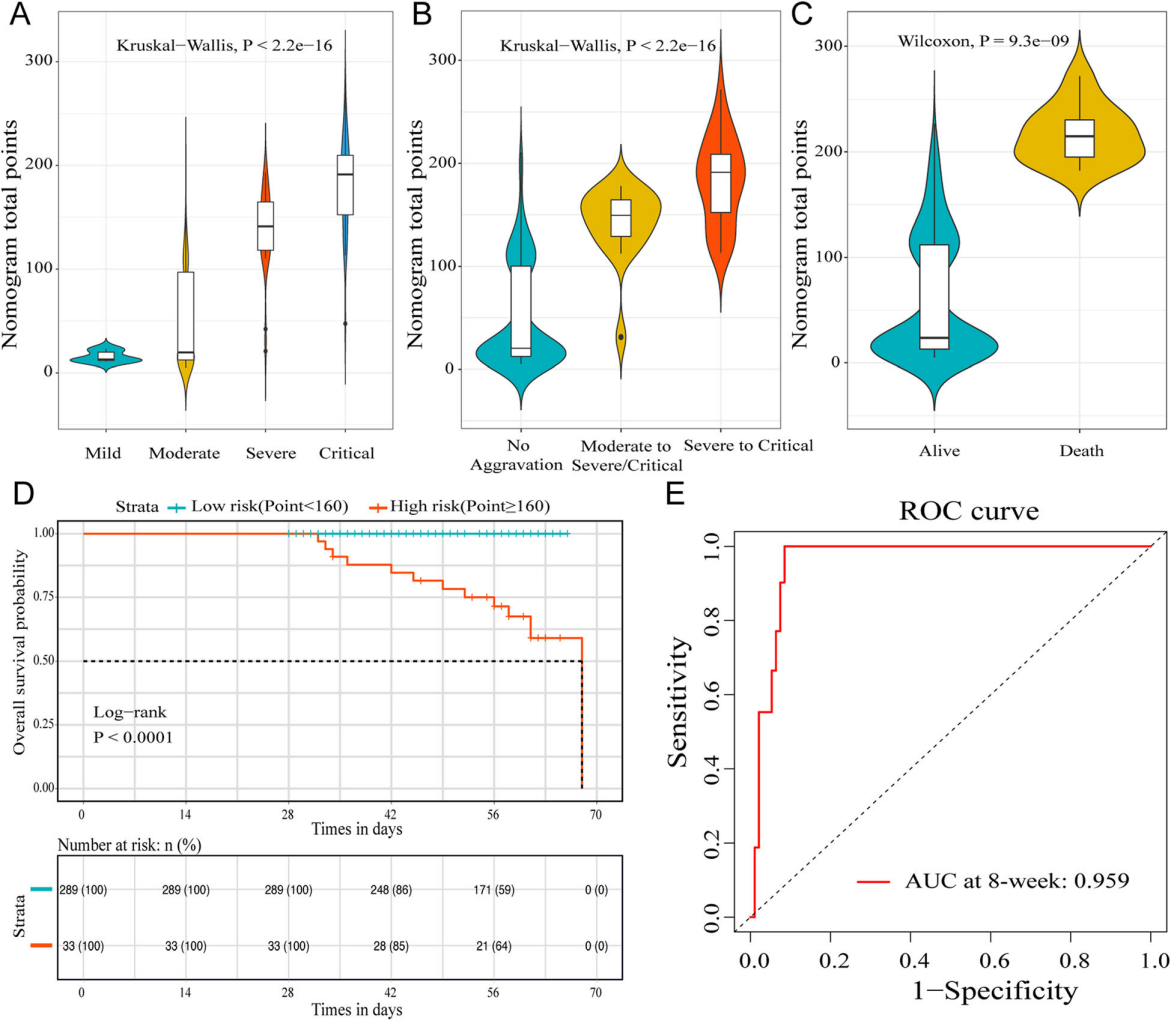
Nomogram for predicting the deterioration and the survival of COVID-19 patients in the total cohort (*n* = 322). **a** Violin plot showing nomogram total points of different clinical types of COVID-19 patients; **b** Violin plot showing the correlation of nomogram total points of patients with and without deterioration; **c** Violin plot showing nomogram total points of alive and dead COVID-19 patients; **d** Kaplan-Meier survival curves based on the relative low- and high-risk patients divided by the cut-off point (*P* < 0.0001). Low-risk = Total point < 160; High-risk = Total point ≥160; **e** Time-dependent ROC curve analysis of the nomogram predicting survival of COVID-19 patients. Abbreviations: COVID-19, coronavirus disease 2019; ROC, receiver operating characteristic; AUC, area under the curve; DCA, decision curve analysis

The total point of each of the 322 cases in the total cohort was calculated based on the nomogram constructed in the training cohort, except for 29 cases due to insufficient information. The total points of patients increased with disease severity, as shown in Fig. 3a (*P*-value < 0.001). Patients with deterioration had higher points than those without (*P*-value < 0.001, Fig. 3b). There was a significant difference in points between alive and death groups (*P*-value < 0.001, Fig. 3c).

The total point of 160 corresponding to 50% of the probability of disease deterioration on the nomogram was defined as the cut-off point to distinguish the high and low-risk patients. A total of 322 patients in the total cohort were divided into low-risk (total point < 160, *n* = 289) and high-risk (total point ≥160, *n* = 33) groups. The average follow-up time was 56 days. The Kaplan-Meier analysis results indicated a significant difference in overall survival (OS) rates between the high-risk and low-risk group patients. The high-risk patients showed poorer OS than the low-risk patients (8-week survival rate: 71.41% vs. 100%, Log-rank *P* < 0.0001, Fig. 3d). Time-dependent ROC curve analysis showed an AUC value of 0.959 at 8 weeks (Fig. 3e), indicating outstanding performance for survival prediction.

## Discussion

COVID-19 has various clinical manifestations from asymptomatic diseases to pneumonia and life-threatening complications. During clinical diagnosis and treatment of COVID-19, some patients may have rapid disease deterioration and even die [19]. Presently, there is no therapy for COVID-19 patients. Therefore, it is important to identify risk factors early to predict the likelihood of disease deterioration, thus reducing mortality [12].

Univariate logistic regression analysis identified 24 variables related to the COVID-19 deterioration and the LASSO regression method was used to construct the prediction model. LASSO regression selects the best prediction subset from the high-dimensional original dataset [14], thus significantly improving the accuracy of predicting the deterioration risk of COVID-19 patients in this study. A simple nomogram with four clinical common predictors, including WBC, CRP, whether Lym ≥ 0.8 × 10^9^/L, and LDH ≥ 400 U/L was developed based on LASSO regression. Liang et al. [12] found that lower lymphocyte and higher LDH are independent factors for predicting the occurrence of critical illness and the CALL study used a sample size of 208 patients [11] and found that lower lymphocyte and higher LDH are independent factors for predicting disease progression, similar to this study results. The AUC of this nomogram was 0.945, which was higher than that of the CALL score (AUC = 0.909), indicating the outstanding performance of the nomogram for prediction. Notably, instead of analyzing the absolute value of lymphocytes and LDH as continuous variables, they were divided into binary variables using clinical experience. Furthermore, WBC and CRP were included in the final risk model as inflammation gauges. Importantly, this nomogram using simple and common clinical features can help clinicians to make quick and better decisions.

The nomogram had excellent discrimination, with an AUC of 0.945. Therefore, this nomogram has outstanding clinical transformation value. The nomogram also showed good calibration, thus a convenient tool with clinical value. Besides, the total points of patients increased with disease severity. COVID-19 patients in the total cohort were divided into high-risk and low-risk groups with a significant difference in survival rate to further assess the performance of the nomogram for predicting survival. The time-dependent ROC analysis suggested that the AUC reached 0.959 at 8 weeks, indicating outstanding performance for survival prediction. Remarkably, patients in the low-risk group were all alive.

However, this study has some limitations. First, this was a single-center retrospective analysis study, thus limiting the generalizability of this nomogram in other centers. Multiple external validations using different settings and populations are required to fully understand the transportability of the nomogram. Second, the patients in this study were collected within the early 3-month period of the pandemic. There were no new diagnosed patients from the same institution used as the validation cohort at the later stage due to the effective management of the epidemic situation by the Chinese government, thus affecting the generalizability of the model. Furthermore, the small sample sizes can affect the interpretation of the study results.

## Conclusion

In conclusion, the simple nomogram showed excellent performance in predicting deterioration risk in COVID-19 patients. It can help to optimize the use of limited resources, especially in areas with several cases and/or shortages of medical resources.

## Supplementary Information

**Additional file 1: Figure 1S** Potential features selection using the least absolute shrinkage and selection operator (LASSO) regression. (**a**) Tuning parameter (λ) selection in the LASSO model used 10-fold cross-validation via minimum criteria. Different mean-squared error (MSE) values were plotted versus log (λ). The numbers across the top of the plot represent the number of features remaining. Dotted vertical lines were drawn at the optimal values using the minimum criteria and the 1 standard error of the minimum criteria (the 1-SE criteria). The optimal λ value of 0.07 with log (λ) = − 2.659 was selected (the minimum criteria). (**b**) LASSO coefficient profiles of the 24 features. A coefficient profile plot was produced against the log (λ) sequence, and the four non-zero coefficients were chosen at the values selected using 10-fold cross-validation

## Data Availability

The datasets analyzed in this study are not publicly available but will be made available from the corresponding author on reasonable request.

## Abbreviations

ALT: Alanine aminotransferase
APTT: Activated partial thromboplastin time
ARDS: Acute respiratory distress syndrome
AST: Aspartate Aminotransferase
AUC: Area under the curve
CHD: Coronary heart disease
CI: Confidence interval
CK: Creatine kinase
CKMB: Creatine kinase-MB
COPD: Chronic obstructive pulmonary disease
COVID-19: Coronavirus disease 2019
CRP: C-reactive protein
CRRT: Continuous renal replacement therapy
CVD: Cardiovascular disease
DBIL: Direct bilirubin
DCA: Decision curve analysis
DM: Diabetes mellitus
HL: Hosmer-Lemeshow
IQR: Interquartile
LASSO: Least absolute shrinkage and selection operator
LDH: Lactate dehydrogenase
NIVV: Non-invasive ventilation
OR: Odds ratio
PCT: Procalcitonin
PT: Prothrombin time
ROC: Receiver operating characteristic
RT-PCR: Reverse transcription-polymerase chain reaction
SD: Standard deviation
TBIL: Total bilirubin
WBC: White blood cells

## References

[1] Cucinotta D, Vanelli M (2020). WHO declares COVID-19 a pandemic. Acta Biomed.

[2] Mahase E (2020). Covid-19: WHO declares pandemic because of "alarming levels" of spread, severity, and inaction. BMJ.

[3] Guan WJ, Ni ZY, Hu Y, Liang WH, Ou CQ, He JX, et al. Clinical characteristics of coronavirus disease 2019 in China. N Engl J Med. 2020:382, 1708–1720. 10.1056/NEJMoa2002032 PMC7092819 32109013

[4] Lai CC, Shih TP, Ko WC, Tang HJ, Hsueh PR (2020). Severe acute respiratory syndrome coronavirus 2 (SARS-CoV-2) and coronavirus disease-2019 (COVID-19): the epidemic and the challenges. Int J Antimicrob Agents.

[5] Xie J, Tong Z, Guan X, Du B, Qiu H (2020). Clinical characteristics of patients who died of coronavirus disease 2019 in China. JAMA Netw Open.

[6] Cao B, Wang Y, Wen D, Liu W, Wang J, Fan G, et al. A trial of Lopinavir-ritonavir in adults hospitalized with severe Covid-19. N Engl J Med. 2020:382, 1787–1799. 10.1056/NEJMoa2001282 PMC7121492 32187464

[7] Grein J, Ohmagari N, Shin D, Diaz G, Asperges E, Castagna A, Feldt T, Green G, Green ML, Lescure FX, Nicastri E, Oda R, Yo K, Quiros-Roldan E, Studemeister A, Redinski J, Ahmed S, Bernett J, Chelliah D, Chen D, Chihara S, Cohen SH, Cunningham J, D’Arminio Monforte A, Ismail S, Kato H, Lapadula G, L’Her E, Maeno T, Majumder S, Massari M, Mora-Rillo M, Mutoh Y, Nguyen D, Verweij E, Zoufaly A, Osinusi AO, DeZure A, Zhao Y, Zhong L, Chokkalingam A, Elboudwarej E, Telep L, Timbs L, Henne I, Sellers S, Cao H, Tan SK, Winterbourne L, Desai P, Mera R, Gaggar A, Myers RP, Brainard DM, Childs R, Flanigan T (2020). Compassionate use of Remdesivir for patients with severe Covid-19. N Engl J Med.

[8] Antinori S, Cossu MV, Ridolfo AL, Rech R, Bonazzetti C, Pagani G, Gubertini G, Coen M, Magni C, Castelli A, Borghi B, Colombo R, Giorgi R, Angeli E, Mileto D, Milazzo L, Vimercati S, Pellicciotta M, Corbellino M, Torre A, Rusconi S, Oreni L, Gismondo MR, Giacomelli A, Meroni L, Rizzardini G, Galli M (2020). Compassionate remdesivir treatment of severe Covid-19 pneumonia in intensive care unit (ICU) and non-ICU patients: clinical outcome and differences in post-treatment hospitalisation status. Pharmacol Res.

[9] Sanders JM, Monogue ML, Jodlowski TZ, Cutrell JB. Pharmacologic treatments for coronavirus disease 2019 (COVID-19): a review. JAMA. 2020:323, 1824–1836. 10.1001/jama.2020.6019 32282022

[10] Cheng Z, Lu Y, Cao Q, Qin L, Pan Z, Yan F, et al. Clinical Features and Chest CT Manifestations of Coronavirus Disease 2019 (COVID-19) in a single-center study in Shanghai, China. AJR Am J Roentgenol. 2020(215):121–6. 10.2214/AJR.20.22959 32174128

[11] Ji D, Zhang D, Xu J, Chen Z, Yang T, Zhao P, Chen G, Cheng G, Wang Y, Bi J, Tan L, Lau G, Qin E (2020). Prediction for progression risk in patients with COVID-19 pneumonia: the CALL score. Clin Infect Dis.

[12] Liang W, Liang H, Ou L, Chen B, Chen A, Li C, Li Y, Guan W, Sang L, Lu J, Xu Y, Chen G, Guo H, Guo J, Chen Z, Zhao Y, Li S, Zhang N, Zhong N, He J, for the China Medical Treatment Expert Group for COVID-19 (2020). Development and validation of a clinical risk score to predict the occurrence of critical illness in hospitalized patients with COVID-19. JAMA Intern Med.

[13] National Health Commission of the People’s Republic of China. Diagnosis and treatment plan of novel coronavirus pneumonia (Trial version Seventh). 2020.

[14] Friedman J, Hastie T, Tibshirani R (2010). Regularization paths for generalized linear models via coordinate descent. J Stat Softw.

[15] Kramer AA, Zimmerman JE. Assessing the calibration of mortality benchmarks in critical care: the Hosmer-Lemeshow test revisited. Crit Care Med. 2007:35, 2052–2056. 10.1097/01.CCM.0000275267.64078.B0 17568333

[16] Vickers AJ, Cronin AM, Elkin EB, Gonen M (2008). Extensions to decision curve analysis, a novel method for evaluating diagnostic tests, prediction models and molecular markers. BMC Med Inform Decis Mak.

[17] Vickers AJ, van Calster B, Steyerberg EW (2019). A simple, step-by-step guide to interpreting decision curve analysis. Diagn Progn Res.

[18] Peduzzi P, Concato J, Kemper E, Holford TR, Feinstein AR (1996). A simulation study of the number of events per variable in logistic regression analysis. J Clin Epidemiol.

[19] Pascarella G, Strumia A, Piliego C, Bruno F, Del Buono R, Costa F (2020). COVID-19 diagnosis and management: a comprehensive review. J Intern Med.

