# Supplementary figures and images for: Development and validation of a simple-to-use nomogram to predict the deterioration and survival of patients with COVID-19
Source: BMC Infect Dis. 2021 Apr 16;21:356. doi: 10.1186/s12879-021-06065-z (PMC8050645; doi:10.1186/s12879-021-06065-z)

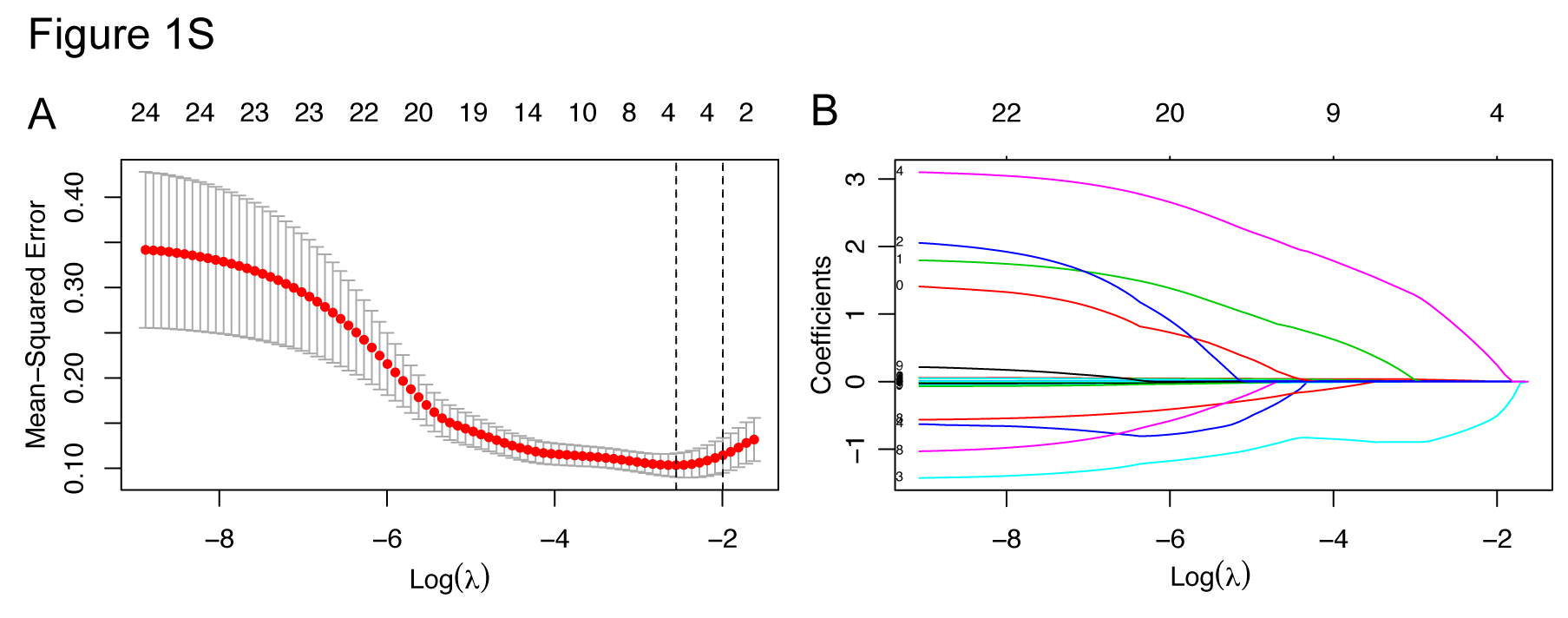

Supplement: Supplementary file 1 — Additional file 1: Figure 1S Potential features selection using the least absolute shrinkage and selection operator (LASSO) regression. (a) Tuning parameter (λ) selection in the LASSO model used 10-fold cross-validation via minimum criteria. Different mean-squared error (MSE) values were plotted versus log (λ). The numbers across the top of the plot represent the number of features remaining. Dotted vertical lines were drawn at the optimal values using the minimum criteria and the 1 standard error of the minimum criteria (the 1-SE criteria). The optimal λ value of 0.07 with log (λ) = − 2.659 was selected (the minimum criteria). (b) LASSO coefficient profiles of the 24 features. A coefficient profile plot was produced against the log (λ) sequence, and the four non-zero coefficients were chosen at the values selected using 10-fold cross-validation [file 12879_2021_6065_MOESM1_ESM.tif]
